# Beneath the Ground: Phylogenetic Position and Morphological Characteristics of *Ityogonimus ocreatus* (Goeze, 1782) (Digenea: Brachylaimidae), a Parasite of European Moles

**DOI:** 10.3390/biology15181643

**Published:** 2026-09-17

**Authors:** Nadezhda Yu. Kirillova, Alexander A. Kirillov, Sergei V. Shchenkov, Elizaveta Yu. Gorodilova

**Affiliations:** 1 Samara Federal Research Center of the Russian Academy of Sciences, Institute of Ecology of Volga River Basin, Russian Academy of Sciences, Togliatti 445003, Russia; nadinkirillova2011@yandex.ru; 2 Department of Invertebrate Zoology, Saint Petersburg State University, St. Petersburg 199034, Russia; svshchenkov@yandex.ru; 3 Research Resource Center for Molecular and Cell Technologies, Saint Petersburg State University, St. Petersburg 199034, Russia; elizaveta.gorodilova@spbu.ru

**Keywords:** Trematoda, Ityogoniminae, *Talpa europaea*, molecular phylogeny, Middle Volga Region

## Abstract

The genus *Ityogonimus* currently comprises three recognized species: *Ityogonimus ocreatus, Ityogonimus lorum*, and *Ityogonimus scalopi*. The type species of this genus, *I. ocreatus*, parasitizes European moles. Despite the widespread distribution of the genus *Ityogonimus,* molecular studies on these parasites have not yet been conducted. The phylogenetic position of this genus remains unresolved. In this study, we present the first molecular phylogenetic data on *Ityogonimus ocreatus* from the European mole in Russia based on partial sequences of its LSU (28S rRNA) gene, alongside a comprehensive morphological and morphometric description of this trematode. Additionally, we obtained sequences of the nuclear ITS2 region and mitochondrial cox1 gene. The morphological characteristics of the flukes examined indicate that they belong to the subfamily Ityogoniminae within the family Brachylaimidae. The molecular data obtained demonstrate that the genus *Ityogonimus* is clearly distinct from other members of the Brachylaimidae. Further comprehensive analyses on brachylaimids, including multilocus approaches, are required to clarify the appropriate classification of the constituent species within the family.

## Simple Summary

The parasitic flatworm *Ityogonimus ocreatus* parasitizes the intestine of European moles. *Ityogonimus ocreatus* is the type species of the genus *Ityogonimus*. Genetic data on trematodes of this taxonomic group are lacking, so the phylogenetic position of this genus remains unresolved. In this study, we collected *I. ocreatus* specimens from the European mole in Russia. We analyzed morphological and morphometric characteristics, provided a detailed morphological description of the parasite, and obtained the first genetic data for this species of helminth. Based on morphological features, *I. ocreatus* belongs to the subfamily Ityogonimiinae within the family Brachylaimidae. Our genetic data demonstrate that the genus *Ityogonimus* is clearly distinct from other members of the family Brachylaimidae. Further comprehensive analyses on brachylaimids are required to clarify the appropriate classification of the constituent species within the family.

## 1. Introduction

The genus *Ityogonimus* Lühe, 1899 currently comprises three recognized species: *Ityogonimus ocreatus* (Goeze, 1782) (=*I. talpae*) and *Ityogonimus lorum* (Dujardin, 1845), which parasitize Palearctic moles [1,2,3], and *Ityogonimus scalopi* Turner & McKeever, 1980, a parasite of the eastern mole *Scalopus aquaticus* Linnaeus, 1758 in North America [4]. The former two trematode species, *I. ocreatus* and *I. lorum*, are common parasites of moles in Europe [1,2,3,5,6,7,8,9,10,11,12,13]. The type species of the genus *Ityogonimus*, *I. ocreatus*, infects the European mole *Talpa europaea* Linnaeus, 1758, the Iberian mole *Talpa occidentalis* Cabrera, 1907, and the Italian mole *Talpa romana* Thomas, 1902 [1,3].

In Russia, the European mole *T. europaea* serves as a host exclusively for *I. ocreatus* among *Ityogonimus*. This species was first recorded in Russia by Wittenberg [14] in moles from the Moscow Oblast. Afterward, this species was found in moles from the Urals [15], as well as the Nizhny Novgorod [16], Tver, Moscow [17,18], and Yaroslavl [19] Oblasts. Despite the widespread distribution of the genus *Ityogonimus,* molecular studies on these parasites have not yet been conducted. Consequently, the phylogenetic position of this genus remains unresolved. In our study, we collected *I. ocreatus* from moles in Russia, analyzed its morphological and morphometric characteristics, and provided the first molecular phylogenetic data for the genus *Ityogonimus*. 

## 2. Materials and Methods

### 2.1. Sample Collection and Morphological Examination

European mole specimens were obtained during a population inventory of myomorph rodents. Trapping was conducted regularly in accordance with the research programs of the Mordovia Nature Reserve, approved by the Ministry of Natural Resources and Ecology of Russia. In July–August 2025, four specimens of the European mole *T. europaea* were incidentally captured across four localities within the Mordovia Nature Reserve: Inorki, Picherki, Plotomoyka, and Srednyaya Melnitsa. Trematodes morphologically identified as *I. ocreatus* were recovered from three of the four examined moles, with a total of 31 digeneans collected.

Only live, mature trematodes were selected for morphological and molecular study. The flukes were isolated from the small intestine of the moles and killed by heating in water. Twenty trematode specimens were used for morphological examination; these were stained with aceto-carmine, dehydrated through a graded ethanol series (70–96%), cleared in clove oil, and mounted in Canada balsam (Panreac, Barcelona, Spain) [20]. For molecular analysis, specimens were fixed in 96% ethanol and stored at +4 °C.

Drawings were prepared using a Levenhuk M500 BASE digital camera (Levenhuk, Inc., Tampa, FL, USA) and an RA-7 drawing tube (Lomo, Saint Petersburg, Russia) attached to an MBI-9 light microscope (Lomo, Saint Petersburg, Russia). All measurements are given in millimeters. Comparative morphometric data were sourced exclusively from published literature containing both illustrations and detailed descriptions of *I. ocreatus*. The digeneans were identified according to Skrjabin [21], Adalid et al. [3], and Petrov and Chertkova [17]. Voucher specimens are stored in the parasitological collection of the IEVB RAS (Togliatti, Russia).

### 2.2. Molecular Data and Phylogenetic Analysis

Total DNA extraction and amplification were performed from a single worm in 50 µL of a 0.05% Chelex-100 (Bio-Rad, Hercules, CA, USA) solution with proteinase K (diaGene, Moscow, Russia). All amplicons were sequenced by “Evrogen” company (Moscow, Russia) using the same primer pairs (for PCR conditions, see Table 1). 

Sequences from both forward and reverse primers were assembled using Chromas Pro v. 1.7.4 (Technelysium Pty Ltd., South Brisbane, Australia). The alignment of partial LSU sequences was created with SeaView 5.1 [27]. The final length of the alignment was 1262 bp. Bayesian Inference (BI) analyses were performed on the 28S rDNA dataset (Table 2). The best-fitted evolutionary model for the phylogenetic analysis was GTR + G + I, as estimated with MrModeltest v. 2.4 [28]. The Bayesian analysis was performed using MrBayes v. 3.2.7a [29] on a local workstation for 15,000,000 generations. The quality of the chains was estimated using the built-in MrBayes tools. Based on the estimates, the first 25,000 generations were discarded for burn-in. The scope of the study was limited to the superfamily Brachylaimoidea. *Liolope copulans* (Liolopidae) was used as an outgroup following phylogenetic reconstruction by Perez-Ponce de Leon and Hernandez-Mena [30].

## 3. Results

### 3.1. Molecular Phylogenetic Analysis

**Figure 1:**
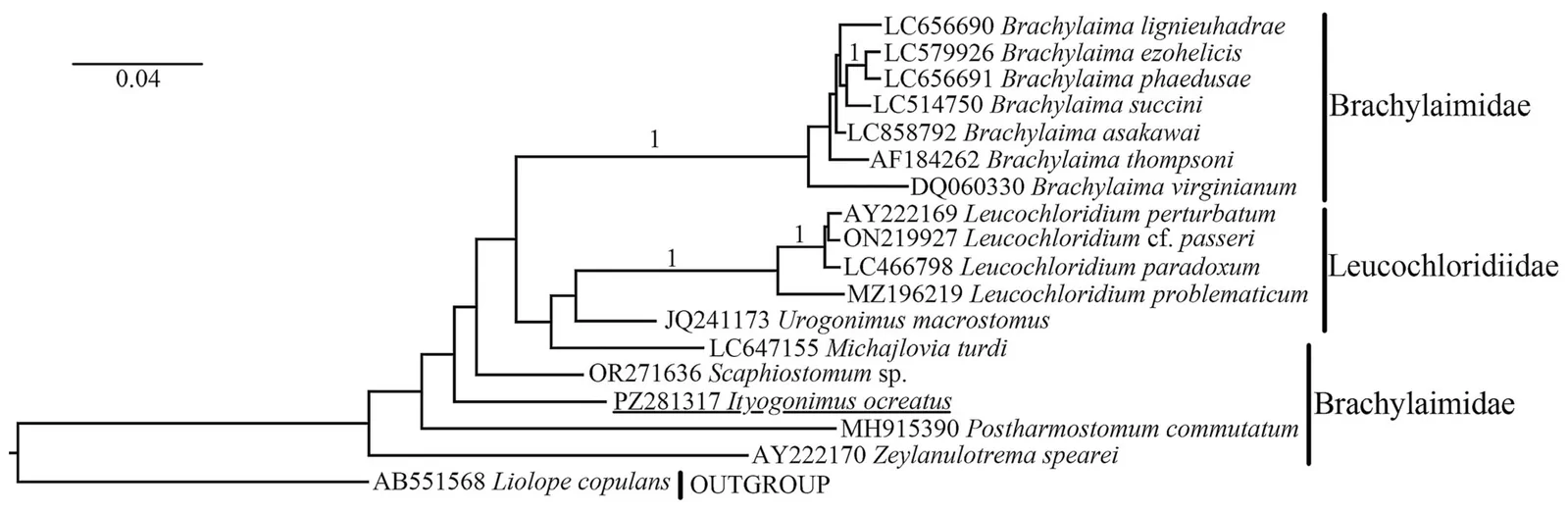
Phylogenetic position of *Ityogonimus ocreatus* inferred from partial LSU sequences. Only significant Bayesian posterior probability values are shown.

*Zeylanulotrema spearei* Cribb & Barton, 1991 (Brachylaimidae) occupied the most basal position among Brachylaimoidea. It was followed sequentially by *Postharmostomum commutatum* (Diesing, 1858), *I. ocreatus*, and *Scaphiostomum* sp. (all of which currently belong to Brachylaimidae). In our analysis, *Michajlovia turdi* (Yamaguti, 1939) (Brachylaimidae) occupied a basal position within the subclade of the family Leucochloridiidae (genera *Urogonimus* Monticelli, 1888 and *Leucochloridium* Carus, 1835), albeit with low support values. Species of the genus *Brachylaima* Dujardin, 1843 formed a well-supported subclade that occupies a sister-group position to the Leucochloridiidae (Figure 1).

In the present study, we obtained sequences of ITS2 but excluded them from the molecular phylogenetic analysis, as the resulting alignments yielded highly inconsistent topologies and, in our view, lacked sufficient informative value for resolving phylogenetic relationships at the level of genera and families within the Brachylaimoidea.

### 3.2. Taxonomy and Morphological Features

Superfamily Brachylaimoidea Joeux et Foley, 1930

Family Brachylaimidae Joeux et Foley, 1930

Subfamily Ityogoniminae Yamaguti, 1958

Genus *Ityogonimus* Lühe, 1899

**Figure 2:**
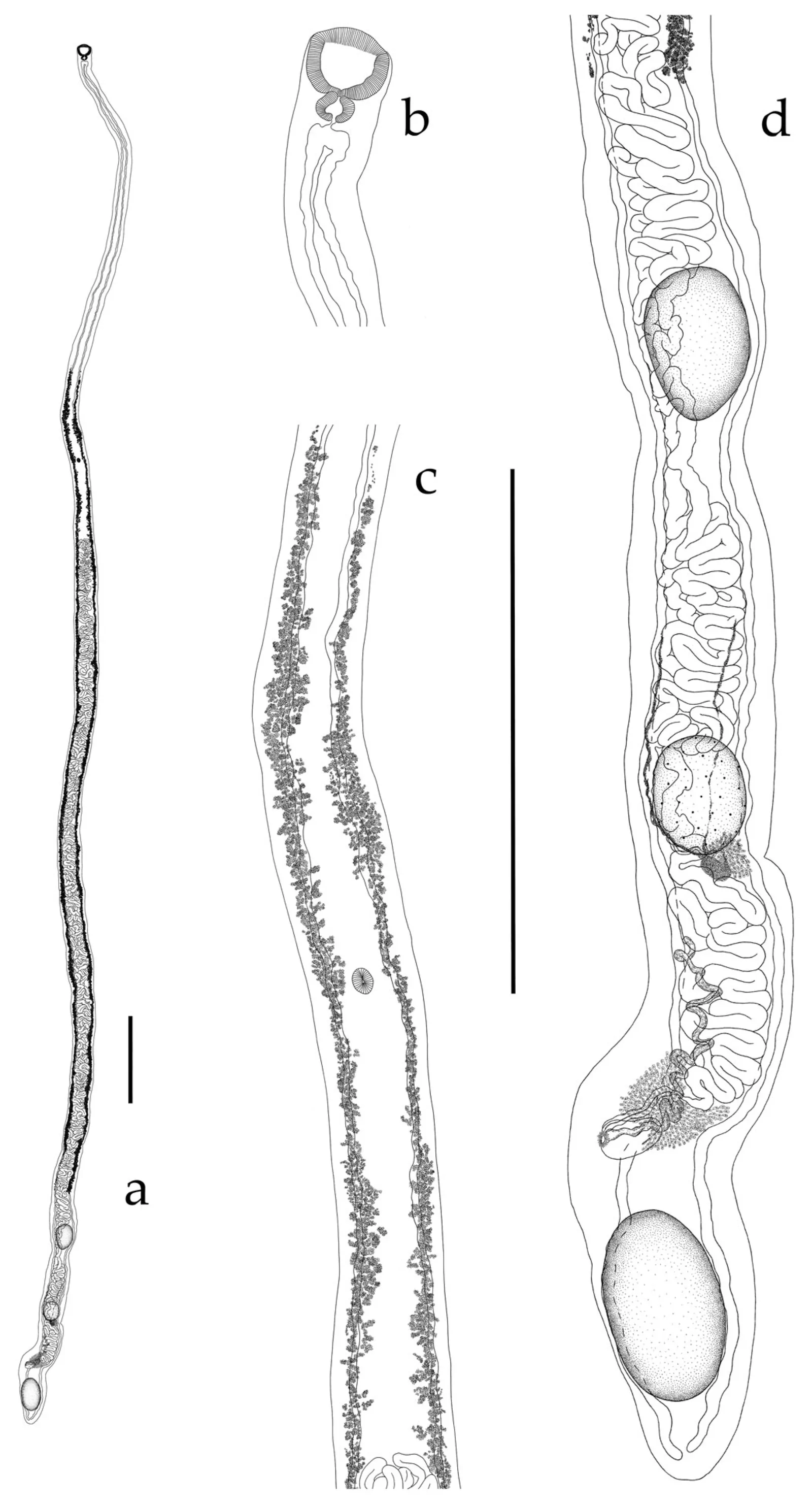
*Ityogonimus ocreatus* ex *Talpa europaea*; (**a**) general morphology (ventral view); (**b**) oral sucker; (**c**) ventral sucker; (**d**) posterior body end. Scale bars = 2.0 mm.

*Ityogonimus ocreatus* Goeze, 1782) (Figure 2)

Host: *Talpa europaea*.

Geographical localities: Mordovia Nature Reserve (Republic of Mordovia)—Inorki Lake (54.727266 N, 43.146248 E), cordon Plotomoyka (54.892595 N, 43.162863 E), cordon Srednyaya Melnitsa (54.903387, N, 43.231963 E).

Prevalence: in three examined moles (75.0%).

Intensity: 3–21 per infected mole (3, 7, 21).

Mean intensity: 7.8.

Availability: GenBank No PZ281317—partial LSU; PZ281315—ITS2; PZ277241—partial cox1.

Accession numbers in the parasitological collection of IEVB RAS: No 2501–2512.

Morphological description of *Ityogonimus ocreatus* (based on 20 specimens; measurements are given in Table 3): Body narrow, elongated, ribbon-like; maximum width in posterior part (Figure 2a). Tegument unarmed. Oral sucker well-developed, terminal (Figure 2b). Ventral sucker round to slightly oval, significantly smaller than oral sucker, situated at approximately the first third of body length (Figure 2c). Pharynx round. Esophagus very short, often indistinct. Intestinal ceca extending along body to posterior extremity. Gonads median, at hindmost posterior end of body. Testes smooth-margined, oval, elongated (Figure 2d). Genital pore at anterior margin of posterior testis, opening ventrally, surrounded by muscular sphincter. Cirrus sac small, anterior to posterior testis, surrounded by numerous glandular cells, containing only ejaculatory duct and cirrus. Seminal vesicle long, convoluted, lying free in parenchyma. Ovary roundly oval, intertesticular (Figure 2d). Mehlis’ gland dorsal, immediately adjacent to the posterior of the posterolateral margin of the ovary, about half of ovary size. Vitellarium composed of numerous small follicles; vitelline fields initiating slightly anterior to, at the level of, or slightly posterior to the ventral sucker. Vitelline fields asymmetrical, with left and right fields initiating and terminating at different levels (Figure 2a). Vitelline fields extending laterally toward the posterior end, terminating at the anterior margin of the anterior testis or slightly short of it. Vitelline ducts originating from posterior follicles, merging behind Mehlis’ gland to form a small vitelline reservoir. Uterus with ascending and descending loops, densely intertwined, occupying entire intercecal space, not extending extracecally. Anterior uterine margin slightly posterior to ventral sucker. Posteriorly, uterine loops extending past the ovary, reaching the anterior margin of the posterior testis. Metraterm well-differentiated. Eggs elongated-oval, light yellow.

## 4. Discussion

Based on their primary morphological characteristics, the collected specimens are assignable to the family Brachylaimidae [43]. Furthermore, a set of distinct features—namely a filiform body, small suckers, the presence of a short esophagus, the uterus not reaching the level of the ventral sucker, and the genital pore positioned between the ovary and the posterior testis—indicates their affiliation with the subfamily Ityogoniminae [43]. According to Pojmanska [43], this subfamily comprises two genera: *Ityogonimus* (parasites of moles) and *Scaphiostomum* (parasites of birds). Taxonomic attributes such as the intertesticular position of the genital pore, well-separated gonads and suckers, and the anterior margin of the vitellaria reaching the ventral sucker level clearly confirm that the studied specimens belong to the genus *Ityogonimus* [43]. The species of *Ityogonimus* parasitizing European moles (*I. ocreatus* and *I. lorum*) differ distinctly in their key morphometric parameters, including body length, ventral sucker size, sucker ratio, and the distance between suckers [3]. The aforementioned features allow us to assign the studied trematodes to *I. ocreatus*. The characteristic features of this species were consistently observed across all examined specimens. In terms of body shape and the topography of internal organs, our results are in good agreement with previously published descriptions of *I. ocreatus* [3,7,14,16,17]. Morphometrically, the specimens generally correspond to prior records (Table 1).

However, our phylogenetic analyses suggested *I. ocreatus* does not form a monophyletic lineage with the other represented Brachylaimidae (Figure 1). The basal branching of *Zeylanulotrema spearei* and *Postharmostomum commutatum* relative to the *Ityogonimus* and *Scaphiostomum* clades suggests their status as early-diverging brachylaimid lineages. Nevertheless, this issue remains open to question, as analyses of alternative phylogenetic markers yield conflicting tree topologies [22,37,40,41]. The well-supported sister-group relationship between *Brachylaima* and Leucochloridiidae contrasts with the weakly supported basal position of *Michajlovia turdi* within Leucochloridiidae, which warrants further validation, although a close relationship between *Michajlovia* and leucochloridiids was previously demonstrated based on SSU sequence analysis [22]. Our LSU-based hypothesis corroborates the non-monophyly of the Brachylaimidae as currently circumscribed [41]. These results necessitate a taxonomic revision of taxonomic boundaries and emphasize the need for combined SSU and LSU markers to resolve deeper nodes. If these findings are consistently reproduced in alternative analyses and across different markers, the paraphyly of the family Brachylaimidae will be robustly confirmed.

## 5. Conclusions

In this study, we present the first molecular phylogenetic data on *Ityogonimus ocreatus* from European moles in Russia and provide morphological and morphometric descriptions of this parasite. The morphological features of the examined trematodes indicate their affiliation with the subfamily Ityogoniminae within the family Brachylaimidae. The analysis of molecular data obtained herein demonstrates that the genus *Ityogonimus* is distinctly separated from other members of the family, although its precise phylogenetic position remains unresolved. Further comprehensive analyses on brachylaimid trematodes, including multilocus approaches, are required to clarify the appropriate classification of the constituent species within the family.

## Figures and Tables

**Table 1:** Primers and PCR conditions used in this study.

Marker	Forward Primer/ Reverse Primer	PCR Conditions	References
cox1, partial	JB3/JB4.5 (Evrogen, Moscow, Russia)	Initial denaturation—95°, 3 min; denaturation—95°, 20 s; annealing—52°, 30 s; extention—72°, 45 s; final elongation—72°, 5 min; 40 cycles	Bowles et al. [22]
ITS2	3S/ITS2.2 (Evrogen, Moscow, Russia)	Initial denaturation—95°, 5 min; denaturation—95°, 30 s; annealing—61°, 30 s; extention—72°, 1 min; final elongation—72°, 5 min; 40 cycles	Cribb et al. [23], Morgan&Blair [24]
LSU, partial	LSU5/1500R (Evrogen, Moscow, Russia)	Initial denaturation—95°, 3 min; denaturation—95°, 20 s; annealing—56°, 30 s; extention—72°, 1 min 20 s; final elongation—72°, 5 min; 35 cycles	Lockyer et al. [25], Van Steenkiste et al. [26]

**Table 2:** Sequences used in molecular phylogenetic analysis.

GenBank No	Trematode Species	Host	Geographical Region	Reference
LC656690	*Brachylaima lignieuhadrae*	*Euhadra dixoni* Pilsbry, 1900 (Gastropoda, Camaenidae)	Japan	Waki et al. [31]
LC579926	*Brachylaima ezohelicis*	*Corvus corone* Linnaeus, 1758 (Aves, Corvidae)	Japan	Direct submission
LC656691	*Brachylaima phaedusae*	*Zaptyx buschii* Küster, 1844 (Gastropoda, Clausiliidae)	Japan	Waki et al. [31]
LC514750	*Brachylaima succini*	*Succinea lauta* Gould, 1859 (Gastropoda, Succineidae)	Japan	Nakao et al. [32]
LC858792	*Brachylaima asakawai*	*Vulpes vulpes* schrencki Kishida, 1924 (Mammalia, Canidae)	Japan	Hidaka et al. [33]
AF184262	*Brachylaima thompsoni*	*Blarina brevicauda* Say, 1823 (Mammalia, Soricidae)	USA	Tkach et al. [34]
DQ060330	*Brachylaima virginianum*	*Didelphis virginiana* Gill, 1872 (Mammalia, Didelphidae)	USA	Direct submission
AY222169	*Leucochloridium perturbatum*	*Turdus merula* Linnaeus, 1758 (Aves, Turdidae)	Czech Republic	Olson et al. [35]
ON219927	*Leucochloridium* cf. *passeri*	*Succinea* sp. (Gastropoda, Succineidae)	Taiwan	Chiu et al. [36]
LC466798	*Leucochloridium paradoxum*	*Succinea lauta* Gould, 1859 (Gastropoda, Succineidae)	Japan	Nakao et al. [37]
MZ196219	*Leucochloridium problematicum*	*Fulica americana* Gmelin, 1789 (Aves, Rallidae)	USA	Direct submission
JQ241173	*Urogonimus macrostomus*	*Passer domesticus* Linnaeus, 1758 (Aves, Passeridae)	Canada	Locke et al. [38]
LC647155	*Michajlovia turdi*	*Turdus cardis* Temminck, 1831 (Aves, Turdidae)	Japan	Sasaki et al. [39]
OR271636	*Scaphiostomum* sp.	*Heteromys gaumeri* Allen & Chapman, 1897 (Mammalia, Heteromyidae)	Mexico	Panti May et al. [40]
PZ281317	*Ityogonimus ocreatus*	*Talpa europaea* Linnaeus, 1758 (Mammalia, Talpidae)	Russia	This study
MH915390	*Postharmostomum commutatum*	*Gallus gallus* Linnaeus, 1758 (Aves, Phasianidae)	Brazil	Valadão et al. [41]
AY222170	*Zeylanulotrema spearei*	*Rhinella marina* Linnaeus, 1758 (Amphibia, Bufonidae)	Australia	Olson et al. [35]
AB551568	*Liolope copulans*	*Semisulcospira libertina* Gould, 1859 (Gastropoda, Semisulcospiridae)	Japan	Baba et al. [42]

**Table 3:** Measurements of *Ityogonimus ocreatus* from the original description and redescriptions.

Characteristic	This Study	Wittenberg [14] ^1^	Morozov [16]	Petrov & Chertkova [17]	Edelenyi [7]	Adalid et al. [3]
Host	*Talpa europaea*	*T. europaea*	*T. europaea*	*T. europaea*	*T. europaea*	*T. occidentalis*
Locality	Mordovia Nature Reserve, Russia	vicinity of Moscow, Russia	Nizhny Novgorod Oblast, Russia	Belarus, Russia	Borzsony, Hungary	Asturias, Spain
Body length	15.1–37.2 (25.6)	19.3–21.8	31.0–43.5	17.0–28.0	17.963	14.044–16.996 (15.022)
Body width	0.436–0.835 (0.610)	0.48	0.466–0.899	0.560–0.717	0.590	0.712–0.970 (0.848)
OS width	0.255–0.456 (0.348)	0.35–0.41	0.265–0.424	0.340–0.369	0.276	0.307–0.445 (0.352)
OS length	0.200–0.405 (0.310)			0.260–0.369		
VS width	0.068–0.109 (0.093)	0.10–0.12	0.106–0.217	0.102–0.123	0.084	0.067–0.125 (0.106)
VS length	0.068–0.127 (0.095)			0.105–0.123		
Pharynx	0.132–0.254 (0.164) ^2^	0.15–0.17	0.106–0.212	0.140–0.164	–	0.135–0.168 (0.151)
Anterior testis length	0.382–0.682 (0.535)	0.50–0.67 × 0.25–0.27	0.636–0.954	–	0.280–0.530	0.374–0.573 (0.470)
Anterior testis width	0.191–0.400 (0.289)		0.318–0.583	–		
Posterior testis length	0.427–0.800 (0.574)		0.580–1.094	–		0.464–0.576 (0.520)
Posterior testis width	0.209–0.473 (0.349)		0.318–0.901	–		
Cirrus sac length	0.136–0.264 (0.199)	0.19	–	–	–	0.086–0.176 ^2^
Cirrus sac width	0.100–0.182 (0.129)	0.15	–	–	–	
Ovary length	0.200–0.500 (0.337)	0.037–0.44	0.318–0.530	–	0.254	0.341–0.454 (0.382)
Ovary width	0.155–0.400 (0.242)	0.31–0.35		–		
Eggs length	0.025–0.038 (0.032)	0.032	–	0.028–0.032	0.027	0.030–0.032 (0.0308)
Eggs width	0.013–0.019 (0.016)	0.016	–	0.012–0.016	0.016	0.013–0.014 (0.0136)
Body length/width ratio	1:33–1:55 (1:43)	–	–	–	–	–
OS/VS width ratio	3.00–4.68 (3.78)	–	–	–	–	2.64–4.75 (3.48)
Distance from OS to VS	6.000–11.392 (7.616)	–	6.24–9.54	5.570–9.280	–	2.910–4.932 (3.846)
Distance between testes	1.518–3.595 (2.162)	–	1.8–3.9	–	–	0.877–1.496 (1.230)

Note: ^1^—cited in Skrjabin [21]; ^2^—diameter; mean values are given in parentheses; OS: oral sucker, VS: ventral sucker.

## Data Availability

The data presented in this study are openly available in Genbank at https://www.ncbi.nlm.nih.gov/genbank/, PZ281317, PZ281315, PZ277241.

## Abbreviations

The following abbreviations are used in this manuscript:

DNA	Deoxyribonucleic acid
PCR	Polymerase chain reaction
IEVB RAS	Institute of Ecology of the Volga River Basin of the Russian Academy of Sciences
OS	Oral sucker
VS	Ventral sucker
BI	Bayesian Inference
ITS2	Internal Transcribed Spacer 2
LSU	Large Subunit Ribosomal
SSU	Small Subunit Ribosomal
